# Comparison of MIBG and Diphosphonate scintigraphy in cardiac involvement of aTTR-FAP

**DOI:** 10.1186/1750-1172-10-S1-P43

**Published:** 2015-11-02

**Authors:** Renata Chequer, Ludivine Eliahou, Hamza Regaieg, Vincent Algalarrondo, Sylvie Dinanian, Dominique Le Guludec, Michel S Slama, Francois Rouzet

**Affiliations:** 1 Bichat Hospital, Nuclear Medicine, 75018, Paris, France; 2 Antoine Béclère Hospital, Cardiology and French Reference Center for Rare Diseases for FAP (Familial Amyloid Polyneuropathy), 92140, Clamart, France

## Background

In familial aTTR amyloid polyneuropathy (FAP) cardiac involvement is of major prognostic value. Two approaches using radionuclide imaging proved relevant in the assessment of aTTR-related cardiac amyloidosis: detection of amyloid deposits with disphosphonates (DPD) and of sympathetic denervation with MIBG. The study aimed to compare the respective value of both approaches in patients with aTTR-FAP with suspected cardiac involvement.

## Methods

We prospectively included 76 consecutive patients identified from the database of the French National Reference Center for Amyloidosis with genetically proven aTTR-FAP (males 62%, Val30Met: 42%, domino-liver transplantation 13%, symptomatic 57%, interventricular septum (IVS)≥12 mm: 52%; left ventricular ejection fraction: 63±10%). They underwent both MIBG and DPD scintigraphy in a delay <3 months. For DPD SPECT, acquisitions were performed 3 hours after tracer injection. Cardiac uptake was visually scored as present or absent and quantified by the ratio between 3D isocount volume of interest generated over the myocardium and a standard volume in lung (H/L). For MIBG, heart-to-mediastinum ratio (HMR) was calculated on planar acquisitions performed 4 h after tracer injection. Cardiac MIBG was classified as normal, mildly, moderately, or severely abnormal.

## Results

The delay between DPD and MIBG 6±12 days. DPD was abnormal in 30 patients (39%) and MIBG in 50 patients (66%; p=0.002). When MIBG was normal (n=26), BS was normal except for 1 patient. When MIBG was abnormal (n=50), BS was normal in 21 patients (42%). The uptake of DPD increased with the denervation score (normal: 0.6±0.2; mild: 0.6±0.4, moderate: 3.4±3.3; severe: 4.5±3.7; p<0.001 between normal and moderate/severe). In patients with a previous domino liver transplantation (n=10), the overall pattern was similar. In asymptomatic patients (n=31), all those with a normal MIBG (n=17) had a normal DPD; MIBG was abnormal in 45% (n=14), 50% had a normal DPD. In addition, HMR was greater (1.8±0.3 vs. 1.6±0.4; p=0.008) and H/L was lower (1.7±2.3 vs. 3.2±3.5; p=0.04) compared to symptomatic patients.

## Conclusion

In patients with suspected cardiac involvement of aTTR-FAP, MIBG was abnormal more frequently than DPD. In particular, DPD abnormalities are delayed compared to MIBG since it was abnormal only when denervation was moderate or severe. In the group of asymptomatic patients, MIBG was abnormal in 45% of patients, and only half of those with cardiac denervation had a positive BS. This suggests that innervation abnormalities as seen with MIBG are more frequent and earlier than significant amyloid deposits as seen with Diphosphonates.

